# Review OSINT tool for social engineering

**DOI:** 10.3389/fdata.2023.1169636

**Published:** 2023-09-01

**Authors:** Martina Nobili

**Affiliations:** Unit of Automatic Control, Department of Engineering, Universitá Campus Bio-Medico di Roma, Rome, Italy

**Keywords:** open source intelligence, OSINT, social engineering, cyber security, cyber threats

## Abstract

In recent years, we observed an increase in cyber threats, especially social engineering attacks. By social engineering, we mean a set of techniques and tools to collect information about a person or target to extort sensitive information. Such information might be used for (industrial) espionage, to blackmail the user, or represent the starting point to perform malicious cyber attacks against the individual or, more often, against the organization they work for. The human factor is often the most vulnerable element in the security of any system, and the mass of information we disseminate online largely facilitates social engineering activities. To prevent and mitigate social engineering attacks, Open Source INTelligence (OSINT) techniques and tools can be used to evaluate the level of exposition of an individual or an organization. OSINT is the collection of information through open sources, that is, sources not protected by copyright or privacy. The article reviews the main OSINT tools for countering and preventing social engineering attacks. Specifically, it proposes the different tools diving them accordingly to the specific information they allow to track (e-mail, social profiles, phone numbers, etc.).

## 1. Introduction

In the recent years, we have seen increased access to online resources and the development of more and more services on the network. This phenomenon, fostered by the COVID-19 pandemic, has increased data production and the growing exposure of each of us on the network. Added to this is an intensifying use of social networks, often related also to work activities. All this has simplified our lives, making many activities in different contexts more flexible, avoiding a lot of physical travel, and allowing more cost-effective management of our time. At the same time, however, it exposes us to greater dangers and risks of a different nature. The number of cyber-attacks increases rapidly. Only in 2021, the average number of cyber-attacks and data breaches increased by 15.1% from the previous year (Forbes, 2023). The data stolen through these attacks is increasingly sensitive and puts the security of individuals and organizations at risk. Moreover, attack techniques have become more and more complex and structured. At the same time, several “user-friendly” tools, often free, are available on the internet (e.g., the SET tool in Kali Linux, 2023). As a result, an increased number of cyber attacks is targeting small enterprises and professional firms with fewer defensive capabilities (Kaspersky, 2023). The main strategies of these attacks are phishing and setting up fake websites to steal the data of users who try to use them.

In particular, the most used attacks vector is ransomware which generally exploits social engineering strategies to perform malware delivery. Social engineering means collecting information about a person's behavior to extort sensitive information and perform malicious actions against the individual or organization for which they work. Such information might be used for (industrial) espionage to be sold on the dark web or to blackmail the user. Nevertheless, they might represent the starting point to perform malicious cyber actions against the individual or, more often, against the organization for which he or she works. These stolen information can lead to the execution of a sophisticated cyber-attack.

This attack greatly impacts the company in terms of cost and reputation. On average, a worldwide data breach costs approximately 4.35 million dollars, a figure that rises to 9.44 million dollars in the United States. In particular, the economic impact is more severe in the healthcare sector, costing approximately 10 million dollars per attack (IBM, 2022). These figures then do not consider the impact of these types of attacks on citizens' lives. An example of these attacks and their consequences for citizens is the attack on the Lazio region healthcare system in Italy in August 2020. The attack resulted in a halt of the administration of the patient care system as well as a slowdown in the vaccination campaign that was taking place (Regione Lazio, 2020).

There are different types of social engineering attacks that can be classified, taking into account various aspects (Salahdine and Kaabouch, 2019). Nevertheless, they have a common pattern of execution that starts with the acquisition of meaningful information about the victim (target) and the subsequent connection with it. Hence, it is crucial for the attacker to collect relevant information about the victim to know personal details, passions, lifestyles, and what is useful to foster a “link” with them to carry out the attack. The nature of information of interest to the attacker depends on the type of attack being carried out. The latter may interest the individual, the organization of the company in which they operate or other elements relating to the work or private context that can be exploited, for instance, to construct a targeted spear phishing attack. For an attacker, there are different strategies to perform such a task. Moreover, the channels used to conduct a social engineering action can include emails, instant messages, social networks messages, and telephones (Krombholz et al., 2015).

One approach increasingly used by attackers is to search inside the so-called open sources, exploiting Open Source INtelligence (OSINT) (Ariu et al., 2017). OSINT can be defined as “the intelligence discipline that pertains to intelligence produced from publicly available information that is collected, exploited, and disseminated promptly to an appropriate audience to address a specific intelligence and information requirement” (USA Headquarters Department of the Army, 2012). Thus, it represents the discipline of intelligence gathering on data sources not covered by privacy or copyright exploiting techniques able to retrieve information 'left behind,' either voluntarily or through carelessness, by a user on the internet or social media.

OSINT has been used since the early 20th century, relying on research from traditional sources such as newspapers. From the birth of the Internet, this discipline has experienced ever greater development and growth (Hassan and Hijazi, 2018). This spread and growth are closely linked to the increased availability of data on the net, which is very often freely accessible.

Several examples in the literature of attacks based on OSINT techniques to steal sensitive data exist. Khanna et al. (2016) analyze how to subtract and elicit personal information through this methodology. Using the Maltego tool (Maltego, 2023) and its extension allows the collection of data inherent to specific targets, such as email, social profiles, profiles linked to the specific email address, and phone number. Possible countermeasures to this type of attack are highlighted in the study. Another study Uehara et al. (2019) shows how starting from an email sent to a subject X, numerous data related to the subject can be inferred. To this end, they integrate the results of Maltego with Twitter search with a specific tool, as Tinfoleak (Tinfoleak, 2023). Related to the use of Twitter, another example of using this source is given by Hoppa et al. (2019). In this study, a pipeline is developed for automated data collection, represented by tweets. Another threat comes from password theft, which is still one of the most vulnerable elements in the security system as it is closely related to the human factor. The study Kanta et al. (2020) shows how using OSINT techniques can speed up the collection of information on subjects. In this case, it was carried out in a “positive” way, going to provide additional support to police investigations. However, this does not exclude the use of the same techniques maliciously, thus threatening an organization's security and integrity.

OSINT can be also used to collect files protected by cryptographic algorithms. Using several techniques (see for example Mozaffari-Kermani and Reyhani-Masoleh, 2011a,b), a malicious attack can crack the protection of inadequate algorithms or keys and consequently diffusion of the stand information. In this way, the attack can acquire more sensible information. Notice that while the simple collection of files as the explanation of the meta-data can be considered an open all, the crack of such file violates the OSINT framework because it cannot be considered as a “gray source” activity violates user rights and law in several countries. However, this cannot be considered as a barrier for a malicious actor; hence, it is mandatory to use free available files with strong cryptographic algorithms (Mozaffari-Kermani and Reyhani-Masoleh, 2009).

Notice that OSINT can also be used to contrast social engineering attacks. Indeed, understanding what kind of data is exposed on the network is extremely relevant to design appropriate awareness campaigns (Assenza et al., 2020). Hence, there are several examples of integrating the OSINT methodology into the organization's security system, particularly in the cybersecurity field. Lande and Shnurko-Tabakova (2019) analyzed integrating OSINT within a cyber defense system and highlighted the key benefits of this practice for an organization, such as cost reduction, effectiveness, and data volume. The article highlights methodology and techniques to integrate existing resources through OSINT. Hayes and Cappa (2018) presented an example of a risk assessment conducted on a target company using only OSINT sources and tools. They identified vulnerabilities present in the corporate network. Moreover, in their investigation, they were able to identify the personal information and opinions of the employees. It emerges how critical it is to adopt specific security policies to avoid disseminating potentially sensitive information and the need to activate proactive OSINT-based initiatives to reduce the risk of accidental information leakage. There are also attempts to integrate OSINT techniques in security standards, as suggested in AlKilani and Qusef (2021), where OSINT techniques are used to assess companies' compliance with ISO 27001.

### 1.1. Contribution

This study reviews available OSINT tools for performing social engineering activities. We considered tools for the collection of information, analyzing some of the most popular and widely used tools, presenting their features and limitations. We provided an overview of which kind of information and/or vulnerabilities can be collected using such methods to provide an instrument to understand the level of exposition and, consequently, define adequate protection initiatives. Consequently, we mainly focus on data relevant to arrange social engineering attacks such as email, username, phone number, and social profile.

The article is organized as follows. Section 2 presents the OSINT methodology. Section 3 illustrates the different social engineering tools divided into five application groups based on the type of information elicited using the specific tool. Section 4 presents an experimental validation of the tools. Section 5 collects some consideration and possible future studies, while Section 6 reports the study results and relevant conclusions.

## 2. OSINT methodology

As discussed above, in the last years, an exponential increase of data available on the web has been observed. Hence, in parallel, the relevance of methodologies and tools able to help users to retrieve valuable information from this huge amount of data has also grown. In this context, OSINT represents an effective methodology to search, collect, analyze, purge data, and potentially exploit relevant information. Doing an OSINT investigation, there is a risk of finding too much inaccurate information that generates noise and not the correct result. These “false positives” and searches are being often carried out quickly, and there can be bias and confusion in the results.

For this reason, it is fundamental to define a correct plan on how to proceed in an OSINT investigation. The first step is represented by the information's research. It is generally based on the research of keywords and analysis of images. Then one has to perform correlation among data to refine the information. And finally, such information needs to be exploited to perform the intended task.

Then, a process to manage the OSINT process. We can define a four-step process:

1. Direction: The first step of the process. It is based on the determination of information needs. It consists of defining objectives, appropriate sources, and choice of time frames. This is the less automatized phase and largely depends on the research experience. Notice that this can be considered the most critical phase because any mistake in the definition of the relevant questions or deficiencies in the data source may produce dramatic consequences in terms of output quality.
2. Collection: It constitutes the second phase of the activity. In this step, data are collected from the identified sources. This is generally performed using bots or scrapers.
3. Elaboration: Intelligence analysis is performed on the collected data. Such tasks are devoted to integrating the information provided by the different sources and identification of data incoherence and lack of information. This process is generally divided into two sub-stages: aggregation and evaluation. In the first sub-stages, data are grouped into interrelated information. In the second stage, such information is evaluated in terms of two basic parameters: the source's reliability and the news's truthfulness. Notice that the reliability of a source does not automatically make the news true. In addition, the fact that a source has provided true news does not make it to be considered reliable.
4. Exploitation: Here, we consider the "use" of the information to perform the intended task. If the processing should not be completed to avoid delay, it should be indicated. While with appropriateness, one must respond to the user's requests and be accessible and understandable to them.

The definition of these steps appears to be common to the different investigations. Nevertheless, it may occur in various forms depending on the contexts and the analyst's choices, but the step nature is common and available in literature (Hwang et al., 2022). The methodology is adapted to the needs and demands of the research, as in the case (Lee and Shon, 2016) where a framework for information gathering in critical infrastructure is presented. Another method to integrate open sources is presented in Pastor-Galindo et al. (2020). In this case, the OSINT cycle is integrated with the DML model representing abstraction levels in cyber attack detection.

The general framework can be tailored to the aim of this article, considering how different resources can be used to collect information with the OSINT methodology.

1. Direction: In this, we should consider and analyze investigation's aim and the type of resources. We can consider the *search engine, email, username, phone number, and social network*.
2. Collection: In this phase, we use the selective source to obtain the raw data about the target of the investigation.
3. Elaboration: After collecting the data, it is important to analyze the output of the previous step to obtain valuable information. To this end, the data are generally aggregated and combined to elicit information. Techniques to analyze the data are not discussed in this article.
4. Exploitation: In this phase, it is essential to produce a report or a document where the investigation results are presented.

**Figure 1:**
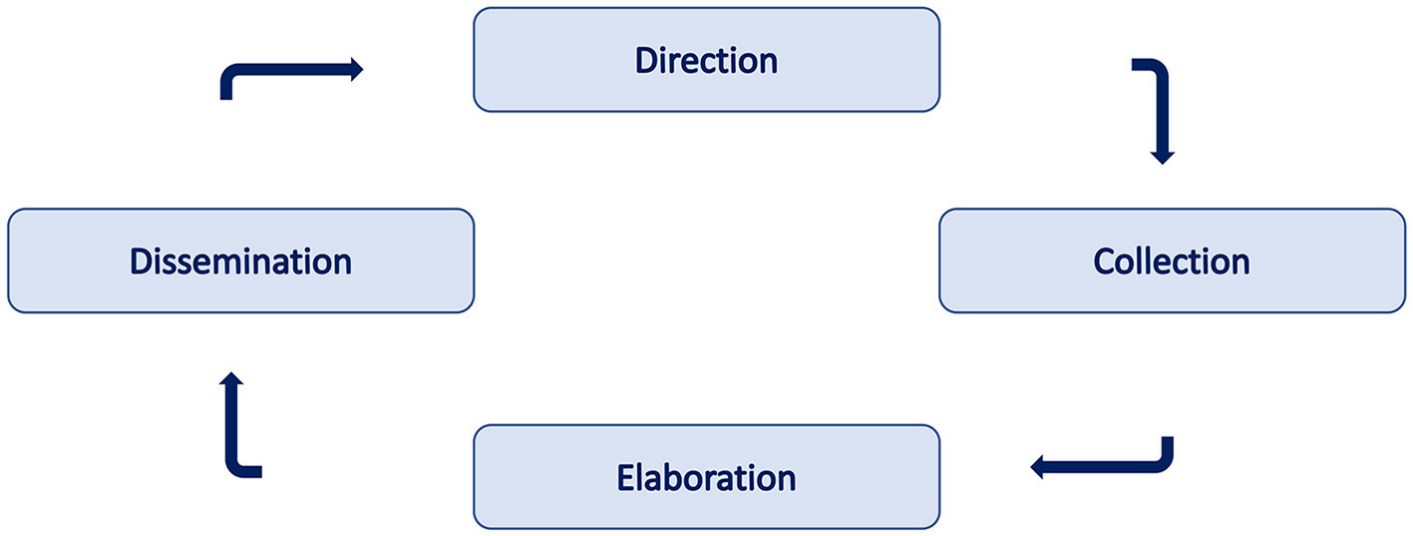
Summary of OSINT circle to collect information.

Figure 1 presents the different phases of research information.

As mentioned earlier, the study focuses on information gathering. To carry out this phase, it is essential to define the steps to be performed and followed. It is possible to define the structural information collection flow:

1. *Target*. The first aspect is to identify the target of the attack, typically a company. After that, it is necessary to search for the information related to it. In this phase, the collection of information is performed on different types of information sources, but it is started by using a search engine. The aim of this step is to collect data about the targets, their structure, and their characteristics.
2. *Company*. After researching possible targets and identifying a company of interest, information about it is sought in order to perform the attack. Information is first sought on search engines even if preliminary information has already been identified. At this stage, it is important to identify the company structure, how it works, and how it communicates.
3. *Employees*. Starting from the company's information, an attempt is made to identify the employees. On the base of the specific attack strategies, it should be of interest to identify specific hierarchies inside the organization, e.g., chief financial officer, IT responsible, and legal. In this activity, different types of tools could be helpful, i.e., email identification and social networks.
4. *Employee information*. After the identification of an employee, it begins the research of information about them. All possible information about them and their lives is sought. One tries to identify and understand their interests and relationships to generate a targeted attack on them.

**Figure 2:**
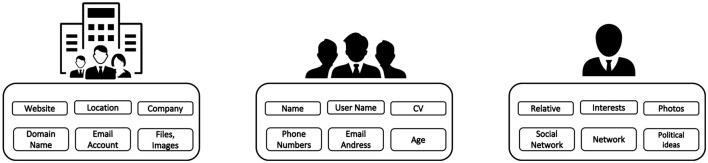
Different collection steps of information to perform an attack.

From the structured collection of this type of information, an attack toward a possible target can be defined and carried out. Hence, this information and structure should be protected to prevent social engineering attacks. In Figure 2, the main information to be collected is summarized and divided into the macro-groups highlighted above.

## 3. Review of the OSINT tool

This section analyzes some of the most common and effective OSINT tools to collect personal information. We focus on tools able to support the performance of social engineering attacks and, conversely, helpful to understand and control the information exposed on the Internet to identify potential vulnerabilities to the security of a company or an individual. We divided this section into five subsections, each focused on tools designed to collect information about emails, usernames, phone numbers, and social networks.

Exploiting the OSINT tools described in this section allows one to elaborate and collect the information illustrated in Section 2. The order in which they are presented follows the structure of the research, starting from the elements that are easier to acquire and then used as a starting point for further refinement and enrichment of information about the target.

### 3.1. Search engine

One of the most useful and used OSINT tools, and the very first starting point for any information collection campaign, is the search engine. They are used by everyone every day to realize textual queries on the web. However, these tools generally provide many answers, that are not always completely reliable. This represents the main criticality of this class of tools. Hence, it is important to carry out targeted research exploiting the features of the tools to refine, circumscribe, and unbias the research results.

*Google* and *Bing* are the most widely used search engines globally. They are certainly the best-performing tools in search depth and the number of indexed sites. Both have filters that allow refinement and more precise and targeted searching by extracting only the information of interest. In this way, the amount of information to be verified is limited in addition to selecting more precise data. Table 1 shows a list of these filters. Nevertheless, both Google and Bing are companies tracking searches and activities the users perform to sell the data. For this reason, both have shortcomings from a privacy perspective.

**Table 1:** Google and bing operators.

**Operator**	**Description**	**Search engine**
" "	It forces the search on the precise term. It is used to avoid ambiguous searches or the use of synonyms	Google, Bing
AND	It returns searches that have both words present in the search	Google, Bing
OR	It returns pages that contain at least one of the keywords entered	Google, Bing
Filetype	It narrows down the results to a certain file type, e.g., pdf, doc, and docx.	Google, Bing
*	Use of wildcard	Google
-	It excludes a word or phrase from the search	Google, Bing
( )	Group words or search operators to control how the search is done	Google
Site	It limits the results to those of a specific site	Google, Bing
Intitle/Allintitle	It returns only pages that have one/all the words specified in the title	Google, Bing
Inurl/Allinurl	It returns only pages that have one/all the words specified in the URl	Google
Intext/Allintext	Returns only pages that have one/all the words specified in the text	Google
Ext	It returns only Web pages with the specified filename extension	Bing
Inanchor/Inbody	These keywords return Web pages that contain the term specified in the metadata	Bing

A lesser-known search engine is the Russian *Yandex*, very popular in Eastern Europe. It has no different features than Google and Bing, also presenting operators that limit language, date, and type of file searched. The main feature of this search engine is that it performs very well in image searches.

Moreover, an interesting search engine is *DuckDuckGo* as this engine does not collect or share users' activities and personal information. Therefore, it can be used to maintain the user's privacy protected. A peculiar characteristic of *DuckDuckGo* is the possibility to use the *bangs !* operator to limit the research on the specific source (e.g., *!tw* limit the research to Twitter).

### 3.2. Email

Nowadays, each company provides an individual email address to each employee. Many have more than one email address not just for their job but also for their personal life. Personal email address is very often associated with online services. Protecting the work email address and the personal one is important because they are largely used for massive cyber-attacks and phishing campaigns. Moreover, if the email of the target is known, it is possible to derive connected profiles and, consequently, personal information about them. People use the same email to register on several websites and, online services and to participate in e-promotion, often using the same password as in their business email. Unfortunately, the level of security of these websites and services is not as high as the business accounts, and it is relatively easy to collect passwords from them. For this reason, emails represent the first step in searching for elements to arrange a social engineering attack. Hence, monitoring how employees spread their email on the Internet is a cornerstone element to be considered in any cyber security strategy.

There are two classes of search tools: look at the domains associated with a company or check known addresses. We present examples for both categories, starting with tools to obtain domain emails.

Hunter (2023) is a web application that allows one to search for the email addresses of a given company. By checking predefined combinations of company addresses, it searches for online correspondence from them. It also allows testing and verifying emails. Through the browser extension, it allows employees' emails to be viewed from the website and thus have the names of employees.

Emailformat (2023) has a list of domains and allows one to search for a specific one and verify its email address composition. It then has a list of email addresses representative of the searched domain with possible verification found on the web. This provides a list of possible employees and places where they used that email. It works very well for US domains but also matches with domains from other nationalities.

Now let us start with a tool to verify an email address and obtain information relative to it. Haveibeenpwned (2023) was born as a web application to verify the compression of an email address after a data breach attack. Moreover, the tool also proves other information that could be stolen during the data breach.

Epieos (2023) is a tool to verify the email addresses. Furthermore, it analyzes how the email is used and shows potential profiles connected to the email. These profiles represent social services or applications where the target has used the same email in the enrollment process. In this way, it is possible to acquire information about the target's interests, communities, and activities. In addition, this tool can return information about the contact, i.e., name, surname, and username.

Email Reputation (2023) is a web tool to verify the reputation of an e-mail address. The tools search the web for any profile or service that uses e-mail. It returns the verification of the email, with a grade of accuracy, and the profile or service associate. It does not come back to the specific profile but gives the existence of a profile in that social.

In Table 2, we report a summary of the tools proposed.

**Table 2:** Tools for OSINT on email address.

**i**	**Tool's name**	**Description**	**References**
1	Hunter	Web application to obtain corporate email	Hunter, 2023
2	Email-format	Tool to search and verify corporate email	Emailformat, 2023
3	Haveibeenpwned	Online application to verify if an email was compromised in a data breach	Haveibeenpwned, 2023
4	Epieos	Tool to verify email address	Epieos, 2023
5	Email reputation	Web application to verify the reputation of an e-mail address	Email Reputation, 2023

### 3.3. Username

One aspect that is generally underestimated but is very relevant in gathering information about a target is the search for usernames. Usernames are names associated with profiles, often related to the target's characteristics or passions. The username alone often does not provide information that can immediately be used to construct a social engineering attack. However, it allows one to determine profiles on social networks or other platforms and, from them, discover unknown email addresses of the target. Investigation of usernames can be performed starting from a tentative to find possible matches. Moreover, it is also possible to start with a username associated with a profile on a specific domain and look for other profiles with the same username.

The first tool for usernames that we analyze is UserSearch. UserSearch (2023) permits searching different profile types starting from a username. It was specifically designed to perform research on social networks, but it can also be used for specific websites and applications. It also has an extension to perform searches on email addresses as well. It returns the profiles associated with the username found on a given platform.

Knowem (2023) has a similar functionality to the previous one. It tests a given username on different types of platforms. In this case, however, it only returns information on whether the name has been used on a determined platform not about the associated profile. Unlike the previous case, however, *KnowEm* performs the search on a greater number and types of platforms.

Another tool for analyzing usernames is NameVine. Namevine (2023) is a tool to analyze usernames on a limited number of platforms. It provides results on whether a match exists on one or more of the analyzed platforms and provides the link to the profile found.

Slightly different as a tool is Leakcheck (2023). This tool allows one to check whether a given username is present within a data breach. It returns as a result the domains that were breached and the date of when this occurred. The results highlighted the presence of data profiles and the actual use of the username.

The latest tool for this section is WhatsMyName. WhatsMyName (2023) is an online tool that allows one to search for a given username on over 500 online platforms. It returns the matches it finds, indicating the type of platform and the link to the identified profile.

In the Table 3 we report a summary of the tools proposed.

**Table 3:** Tools for OSINT on username.

**i**	**Tool's name**	**Description**	**References**
1	UserSearch	Tool that allows to profile starting from username	UserSearch, 2023
2	KnowEm	It checks on different platforms whether a given username has been used	Knowem, 2023
3	NameVine	Tool to analyze the presence of social network profile from a username	Namevine, 2023
4	LeakCheck	It allows to check if a username is inside a data leak	Leakcheck, 2023
5	WhatsMyName	It is a web application that permits to search a username in different domain	WhatsMyName, 2023

### 3.4. Phone number

Private or business phone numbers are largely exploited to perform social engineering attacks. Notice that, in several countries, phone numbers are considered sensible data. It is possible to obtain different types of information about the target from the phone number. Moreover, if one knows the target's phone number, it can be used to directly realize an attack, sending fake messages containing malicious links or malware.

As for the previous case, there are different classes of tools to obtain the phone number, either to retrieve the latter in larger data searches or to retrieve data obtainable from a given phone number. In the first case, the phone number is the object of the research; in the second case, the attacker has discovered the phone number from a different source and he/she wants to associate it with a target to acquire more information.

An example of a tool to elicit the phone number of a target starting from knowing the target's email is Email2phonenumber (2023). As a Python OSINT tool, it permits obtaining the phone number of a target just by having his email. It uses a scraping of different platforms, searching the phone numbers associated with the email.

Syncme (2023) is a tool that allows one to search for a phone number and obtain information about the owner. Specifically, the free version permits only to see the location, the name of the subject, and possible photo. The paid version allows obtaining more information such as the photo of the phone number's owner from social networks and a report of his past activities. This type of information could be very relevant to design sophisticated social engineering attacks.

Another tool that allows obtaining information about a phone number is Phone Validator. Phone Validator (2023) allows to search the phone number of a target and find information about the last location, the type of the number, and the phone company. The paid version allows obtaining also information about the owner. A limitation of this tool is that it is only usable with North American numbers.

The tool Moriarty Project (2023) is a Python tool that allows searching phone numbers. It permits to search for different aspects: the owner of the number, if it has a spam risk situation, possible link connect with the number, and possible social platforms or profiles connected with it.

True Caller (2023) is an OSINT tool to identify whose telephone number it is, whether it is in the name of an individual or a number linked to a company. In addition, this tool makes it possible to search for numbers from different countries.

In Table 4, we report a summary of these tools.

**Table 4:** Tools for OSINT on phone number.

**i**	**Tool's name**	**Description**	**References**
1	Email2phonenumber	Osint tool to obtain a target's phone number from email address	Email2phonenumber, 2023
2	Syncme	Web application that permits obtaining name and photo of the subject from phone number	Syncme, 2023
3	Phone validetor	Tool to obtain information about the phone number	Phone Validator, 2023
4	Moriarty Project	Tool to find a phone number	Moriarty Project, 2023
5	True Caller	Verify real user of a phone number	True Caller, 2023

### 3.5. Social network

Most employees use social networks, even during business time, leaving social media footprints, i.e., trace of the daily activities performed by the user on a social platform. This information can be used by the attacker to perform social engineering attacks. Indeed, from the analysis of the social media footprint, it is possible to understand the habits, the common activity, and the interests of the target. There are different services and tools to collect information about a target from a social network. It is important to underline that a company should adopt specific policies regarding the use of social media by its employees to prevent the spread of sensible data on social platforms.

There are many social networks, each with its own characteristics and peculiarities. Consequently, it is useful to have both tools able to search information on several social networks at the same time (i.e., cross-media search) and also tools designed to be able to extract information from specific platforms, such as Facebook, LinkedIn, and Twitter.

First, we analyze tools to perform cross-media investigations. Social Searcher (2023) is a tool that permits obtaining the social profile from the username or name of a subject. This tool investigates different sources, i.e., Facebook and Instagram. It provides a list of the possible profiles of the subject on each social media.

A similar tool is Webmii (2023). Webmii returns the social profiles associated with a name. In addition, it associates a relative score to the profile, representing the reliability of the result. It associates with the result the sources of the profile, i.e., web and social. It also provides username discovery on the social network. An interesting feature is that it provides information about the people connected on social media with the target. Lastly, it is associated with the Google search engine.

Tools that analyze different social networks provide a broader overview of the analysis. However, they may generate many false positives, i.e., profiles that are not referable to the target. This imposes the user to perform further analysis to check the quality of the results. To partially overcome such limits, it is possible to use tools tailored to research single social networks. In particular, we will look at some of the most widely used social media, i.e., LinkedIn, Instagram, and Twitter. Let us start with Linkedin, a professional social network. It allows one to obtain a wealth of professional information about the target and the work environment and company in which he or she works.

RocketReach (2023) is a web application that knows the name of a target (both an individual or a company), and it allows extracting from LinkedIn information about the target. Starting from the target's name, it gives back the associated profile and possible contact information. Moreover, it verifies the existence of such information on the web. At the same time, starting the research with a company name, it is possible to obtain global information about it, i.e., headquarters address, website, and area of expertise. In addition, employee information and profile are shown. In this way, it is possible to obtain the email address and information about the people that work in the organization.

We now look at Instagram, a social network from the Meta group, where users share photos, videos and activities, often indicating their location.

Pikuki (2023) is a tool that allows one to see Instagram posts without having an account on the social network. It allows searching for a profile without knowing the username associated with it, just enter a first and last name. The system is not always constantly updated, so sometimes it shows posts that have been deleted by the user, and this could provide interesting data. It should be noted that the system allows viewing only posts from public profiles, while for private profiles, the tool is able to provide just the profile photo and the associated username.

Another tool with similar functionality is Pixwox (2023). It is a tool that allows viewing Instagram profiles without having a profile on the platform. The main difference with respect to *Pikuki* is the possibility to download the profile photo of any profile, even private ones. This aspect is very useful when searching for a target because it allows us to extend the search activities also to images. Moreover, it allows viewing the stories saved on the profile as well as making downloads of the posts.

From the Meta group is Facebook, one of the most popular and widely used social networks. Over the years, its popularity and target audience has changed a lot, but it remains a daily diary of many users' activities and thoughts. As a result of some user privacy issues, however, it has undergone many restrictions that led to the shutdown of many OSINT tools designed to perform analysis on this social media. However, accessing its data through some alternative techniques is still possible.

In particular, it is possible to take advantage of some tools that are not really for OSINT use but that allow viewing web pages and, Facebook profiles without logging into the social network or having a Facebook profile. Indeed, one can search for the user's profile by taking advantage of the operators in the search engine section. Once the profile is found, one can test the mobile-friendly mode, which allows one to test viewing a web page on a mobile device. This is usually a feature that is used by developers when building websites or applications. Once it tests the page, it generates the HTML code that describes it. Copying the same to any code viewing tool will result in the page being found. Obviously, with this type of search, navigation on the user's profile is limited, and it is up to the analyst to highlight the information present.

Now, we turn to Twitter, one of the most widely used and popular social networks. Twitter is based on writing short texts expressing one's thoughts on news facts, events, passions, etc.

To analyze Twitter, one can refer to Truth Nest. Truth Nest (2023) is a tool that allows us to get from searching the username of a Twitter profile to find the info about it. As with Instagram, the tool will enable us to analyze and see some tweets without logging in to the platform. In this case, it is necessary to subscribe to the service. The information it returns is varied. First, it provides preliminary information about the profile, such as the name, when and where it was created, and the description that the user has entered. In addition, it provides an overview of the activities performed by the profile and the most popular posts it has made. An interesting feature is a possibility of having information about the profile's network, both people who are followed and those who follow it. Finally, it returns information on how to interact with the profile, such as topics it has talked about. All statistics are collected in a PDF file that can be downloaded.

Continuing TikTok, the platform is a Chinese social network that is becoming increasingly popular among younger people. It is based on making short videos of different themes.

UrleBird (2023) is an OSINT tool that permits visualizing TikTok profiles and videos without an account on the social network. It is similar to the tool presented above for Instagram. The research is possible both by username and by hashtag. The research for hashtags could be very helpful when the username is not known but the activity or the subject of the channels is. Finding a profile, it is possible to see the profile photo and the description in addition to the shared videos.

Finally, let us turn to message applications, i.e., WhatsApp and Telegram, which can be considered social networks and allow us to obtain significant information.

WATools (2023) is a tool to track WhatsApp activities. It permits monitoring the access to the application and the duration of its use of it. It is possible to activate a function that sends a notification when a contact is online and to analyze when a person is connected to the platform. It also allows you to view and download the profile photo associated with the phone number. It can be very useful in verifying an identified phone number and continuing an image search.

Another messaging platform that is becoming increasingly popular is Telegram. Telegram is not just a standard messaging platform, where you communicate between known phone numbers, but it allows also you to create channels to discuss about topics of interest. You can also interact with chatbots. Telegago (2023) is a tool that allows investigating inside the functionality of Telegram. The search is performed by keywords and returns different types of results. First, it provides an overview of the results associated with the topic entered as a keyword, and then it shows the public channels that deal with that topic or have talked about it in posted messages. It also allows seeing contacts involved with that topic, voice chats, and bots. It proves very powerful data if you want to analyze a particular phenomenon or establish a relationship with the target subject of the attack.

In Table 5, we report a summary of the tools proposed.

**Table 5:** Tools for OSINT on social network.

**i**	**Tool's name**	**Description**	**References**
1	Social Searcher	Web application that allows to obtain a social profile from the username or name of a subject	Social Searcher, 2023
2	Webmii	Tools that permit to obtain information about social profiles starting by the name of a subject	Webmii, 2023
3	RocketReach	Web application to search a person or company to obtain the email address and additional information	RocketReach, 2023
4	Pikuki	Tool to search and visualize Instagram profiles without an account	Pikuki, 2023
5	PixWox	Tool to search and visualize Instagram profiles without an account	Pixwox, 2023
6	Truth Nest	Tool to analyzed Twitter profiles from username	Truth Nest, 2023
7	UrleBird	Tool to search and visualize TikTok profiles without an account	UrleBird, 2023
8	WATools	Tool to track WhatsApp activities	WATools, 2023
9	Telegago	Tool to search and analyze Telegram channels	Telegago, 2023

### 3.6. Collective tools

In addition to the tools presented so far, there are instruments that allow to search and analyze different kinds of information from different types of data. These types of tools make it possible to collect amounts of data of different types within a single search.

Maltego (2023) is one of the most well-known and widely used OSINT tools. The system collects and links data from different sources and reports them within a single dashboard via graph. The system is based on two concepts entities and transformations. Entities are represented as nodes in a graph. Investigations begin with one or more entities, on which transformations are performed to explore the relationships between these entities and other yet unknown information. Entities can be of different types-emails, phone numbers, people, in-directories, web domains, etc. Transformations, on the other hand, are pieces of code that, when executed, generate information based on information we already have. Transformations look up information about an entity in the graph and allow an API or database to be queried to show related information in the graph.

Moreover, another tool similar to Maltego that is able to collect data from different types of sources and show it in a graph way is Lampyre (2023). Starting from an input, i.e., email, phone number, or person, it is able to perform research on different sources and show the results in a dashboard. Then, from the results, it is possible to proceed with additional research.

Advantage of this type of tool is the possibility to collect data from one tool without the necessity to switch to different sources. The disadvantage is that it could produce confusion on the results. They produce a lot of results that it is needed to verify.

This type of tool is not comparable with the others for the characteristics they have.

## 4. Experimentation

In this section, we present a test of the different types of tools. The analysis is performed on actual and synthetic data with respect to the different elements enumerated in the previous section. A table summarizing the results obtained is presented at the end.

### 4.1. Email

The experimental test of the email address tools was done in two steps. First, corporate email addresses were searched using Hunter.io and email-format tools, just as one would operate in an actual OSINT search. Five companies from different countries were selected, whose names will not be specified. The names and email addresses of the employees of the selected companies were researched using both tools. Notice that the tools found all but one company, while both Hunter.io and Email-format could not provide information about the last company. Results were then compared to experimentally test their efficiency. The comparison showed greater accuracy and confidence with the results of the Hunter.io tool. The results provided by email-format appear to be not updated, and this may result in multiple false positives.

The found emails were used as inputs for the remaining tools to experimentally check them. Regarding business emails, *Epieos* shows a limitation in finding these addresses. This depends on the tool's structure as it searches for comparison and validation on different platforms and social networks, where there are few business addresses. Emailreputation, on the other hand, recognizes the business emails and provides a high grade of reputation correctly. Regarding the *HaveIbeenPwd* tool, many of the tests performed on the emails were unsuccessful, i.e., it could not find the email. Notice that this means that the companies were not victims of data breaches in the past.

The same tools were tested also for personal emails.

The tool that provided the most positive results, in terms of correct match between email and user, was the Epieos tool. Table 6 shows the results.

**Table 6:** Validation results of OSINT tools on email research. P is positive results, NF is not found, and FP false positive.

**i**	**Subject**	**Tool's name**	
		**Haveibeen pwned**	**Epieos**
1	k**********y@******.com	NF	NF/P
2	a*************z@******.com	NF	NF
3	s***********y@******.com	NF/P	P
4	e*******h@*****.com	P	P/P
5	a**a@*****.com	P	P
6	g**************o@**************.com	NF	NF
7	f************o@**************.com	NF	NF
8	c*************c@******.com	NF	NF
9	m************r@************.com	NF	P
10	o***********f@************.com	NF	P

### 4.2. Username

The validation of usernames was done through two different steps. Ten different usernames were randomly generated through the use of dedicated tools. Then, these usernames were used as an input for the different tools. In this way, it was possible to test their capability to acquire a profile starting from a given username. Notice that a username can be used multiple times by the same user or different users and on different platforms. Matches were sought among the results proposed by the same tool and by comparing the results presented by the other tools examined. In the second phase, a precise match was sought with the target under examination.

The different tools responded well to the tests performed, reporting a nearly 100 percent positive result rate when comparing the results between the different proposed tools. The major limitation at this stage is that one is not researching a person specifically but testing the accuracy of a product, so it cannot be ruled out that in a search for a specific target, the tool would not have a high rate of false positives. This is because they presented different matches on different platforms by testing generic names. Certainly, good reliability of the products emerges, but this must always be accompanied by human analysis to verify the correlation between the results. The presented profiles can be an additional step in information collection, but false positives must be eliminated.

An exception in this discourse is LeackCheck, a tool that verifies the presence of a username within a data breach. The platform had many matches with time-dated attacks and profiles from platforms less used in recent years, which may be a limitation in the use of the tool.

It is possible to see the summarized results in Table 7.

**Table 7:** Validation results of OSINT tools on username research.

**i**	**Subject**	**Tool's name**				
		**UserSearch**	**KnowEm**	**NameVine**	**LeackCheck**	**Whatsmyname**
1	a*******a	P	FP	P	P	P/FP
2	m*****e	P	FP	P	P	P
3	k*****a	P/FP	FP	P	P	P
4	l*****e	P/FP	P	P	P	P/FP
5	a*******o	P/FP	P/FP	P	P	P/FP
6	m*******e	P/FP	P/FP	P/FP	P/FP	P/FP
7	o*****a	P/FP	FP	P/FP	P/FP	P/FP
8	a*****l	P/FP	NF	P/FP	P/FP	P/FP
9	s******t	P/FP	NF	P/FP	P/FP	P/FP
10	f********a	P/FP	NF	P/FP	P/FP	P/FP

P is a positive result, NF is not found, and FP is a false positive.

### 4.3. Phone number

For the phone number, we use a phone number generator available online to generate 10 different numbers to test. The different tools respond with interesting results to the test. For the tool where the geographic spread was limited to one country, we tested only the numbers of that country. It is the case of *Phonevalidator*, which responds with a 10% rate of error, but it covers only the United States, and it reported the geographic area and location of each number identified and if known also number characteristics. For the other tools, numbers from other countries were used in the tests, and good results were obtained. *Sync.Me* obtained an error rate of 30%, showing a preference for finding U.S. phone numbers. In contrast, the Python tool *Moriarty Project* searches on numbers from different countries found only one error, thus an error rate of 10%. The two tools provide almost the same information about the phone number's owner. No tool was able to obtain additional information about the phone numbers. Sync.me, however, showed the associated user's name, while the Moriarty and PhoneValidator tools provided information on the type of number found and the operator associated.

In the second step, the tools were also tested through the phone numbers found in the information search through the other tools. This was done using the same procedure as in a classic OSINT survey. As such, it was possible to get a greater and clearer view of the effectiveness of these tools. During the other phases of the research, three different phone numbers were found, from three different countries, and they were all tested by different tools. Sync.me and TrueCaller showed the best results by associating it with the searched user. Moriarty Project tool was unable to find any of the three numbers. Being PhoneValidator available only for U.S. numbers, testing it with just a single number from this country was possible, and it found a correct match.

The *Email2phonenumber* tool did not match any phone number associated with the emails.

The results were reported in Table 8.

**Table 8:** Validation results of OSINT tools on username research.

**i**	**Subject**	**Tool's name**			
		**sync.me**	**Phone validetor**	**Moriarty project**	**TrueCaller**
1	+1 2********4	P	P	P	P
2	+1 4********4	P	P	P	P
3	+1 9********0	P	P	P	P
4	+1 8********5	P	P	P	NF
5	+1 5********8	NF	FP	P	/
6	+1 3********9	NF/FP	P	P/FP	/
7	+39 3********8	P	/	P	P
8	+44 1*******2	P	/	P	P
9	+46 1********1	NF	/	P	P
10	+1 1********5	P/FP	P	P	P

P is a positive result, NF is not found, and FP is a false positive.

### 4.4. Social network

For social network validation, as in a classic OSINT search, we started from the results obtained in the previous stages, i.e., names and usernames.

First, the tools that simultaneously performed a search and analysis of multiple social networks, Social Searcher and Webmii were analyzed. Regarding the first tool, it has a low success rate, failing to find a match with as many as six of the ten profiles tried. Of the remainder, a success rate of 50%. Webmii, on the other hand, presents more satisfactory results, giving no match in only one case and with a success rate of 70% and with three cases of false positives.

We then moved on to analyze specialized tools. It started with RockReach, which analyzes LinkedIn profiles. This tool is one of the best performing tested, having found 80% of the profiles and with 100% success rate. This tool identifies usernames used later or other analyses.

Two Instagram-specific tools, Pikuki and PixWox, were tested, presenting the same results. In this case, the profiles identified are only 40%, with a 50% rate of false positives. This depends on several factors, such as the presence of homonyms. Only one case, however, yielded no matches. It is difficult to understand whether there is a lack in the tool or if the searched individuals do not have a profile on the social network in question.

Analyzing the TruthNest tool that specifically searches for Twitter profiles, many profiles were absent on the platform. In this case, we can have more confidence that these profiles do not exist as there were no hits on other tools indicating the presence of Twitter profiles. Twitter is also a lesser-used social and used primarily for business communication. Of the matched results, there is a 70% positive rate.

Finally, the tool that searches for and displays TikTok profiles was analyzed. In this case, the validation is biased as the searched profiles are not the platform's target and may most likely not have a profile on the platform. In this case, the false positive rate found is 70%, with only one case found to be positive and two cases of which no correlation was found in the platform.

All the results are presented in Table 9. From it, it is possible to get a summary view of the performance of different social network survey tools, comparing them with each other, and it also allows us to highlight how these results are also influenced by the subjects being researched. In fact, it is evident that some subjects are more prone to activity on social networks, having encountered profiles on almost all platforms, compared to others with almost no profile.

**Table 9:** Validation results of OSINT tools on social network.

**i**	**Subject**	**Tool's name**						
		**Social Search**	**Webmii**	**Rocket Reach**	**Pikuki**	**PixWox**	**Truth Nest**	**UrleBird**
1	k**********y@******.com	P/FP	FP	P	FP	FP	NF	FP
2	a*************z@******.com	NF	P	P	P	P	NF	FP
3	s***********y@******.com	FP	P	P	FP	FP	P	NF
4	e*******h@*****.com	FP	FP	P	FP	FP	NF	FP
5	a**a@*****.com	P	FP	P	FP	FP	FP	FP
6	g**************o@**************.com	NF	FP	P	FP	FP	P	FP
7	f************o@**************.com	NF	P	P	P	P	P	P
8	c*************c@******.com	NF	P	NF	P	P	NF	NF
9	m************r@************.com	NF	P	P	P	P	P	FP
10	o***********f@************.com	NF	NF	NF	NF	NF	NF	NF

P is a positive result, NF is not found, and FP is a false positive.

## 5. Discussion and future studies

The article shows several tools for carrying out a social engineering attack. All these tools can be used both to prepare for an attack and to defend against it. It is important to know them in order to understand one's vulnerabilities and try to protect oneself from possible malicious attacks. It is possible to use the tools presented to perform an assessment of the exposed data and prevent it from remaining so in order to protect yourself from potential threats.

Clearly, it is not possible to identify a tool that provides error-free results. These tools allow us to help in the search for information, but they are not infallible, and it is always necessary to go and verify the information obtained. Several tools have been presented that allow us to obtain different types of data, but it is necessary to combine the different information obtained in order to get an overview of the subject being researched. An overview of the different proposed tools and their error rate and success in correctly identifying the target is given in Table 10. As mentioned earlier, it is not possible to find matches for all the proposed tools and keep in mind that the Maltego and Lampyre tools are not comparable. In addition, tools that provide the structure of emails are also comparable with other results. The tools with the most effectiveness seem to be those for detecting and individuating usernames. It must be kept in mind that the use of certain usernames associated with a user must be verified and are not always used on other platforms as well. The research done and the data required are closely interconnected, and therefore, there is a need for a combination of the information and tools obtained.

**Table 10:** Report an overall of the tools proposed.

**Tool's name**	**Target**	**Results**	
		**Positive rate**	**Error rate**
Hunter	Email	-	-
Email-format	Email	-	-
Haveibeenpwned	Email	30%	70%
Epieos	Email	60%	40%
Email Reputation	Email	-	-
UserSearch	Username	100%	0%
KnowEm	Username	70%	30%
NameVine	Username	100%	0%
LeackCheck	Username	100%	0%
Whatsmyname	Username	100%	0%
Email2phonenumber	Phone number	-	-
Syncme	Phone number	70%	30%
Phone Validetor	Phone number	90%	10%
Moriarty Project	Phone number	90%	10%
True Caller	Phone number	77%	33%
Social Searcher	Social network	40%	60%
Webmii	Social network	90%	10%
Rocket Reach	Social network	80%	20%
Pikuki	Social network	40%	60%
PixWox	Social network	40%	60%
Thruth Nest	Social network	70%	30%
Urle Bird	Social network	70%	30%
WATools	Social network	-	-
Telegato	Social network	-	-

In future studies, we will show how to use these tools to gather this information in order to execute a possible attack.

In addition, you will be able to show added tools to be structured in the other stages of informative collection, such as information analysis.

## 6. Conclusion

The OSINT methodology applied to social engineering attacks was analyzed in practice. The article presented several tools capable of identifying key information of targets sought in these types of attacks. The different resources were classified with respect to the target of a reference and the main characteristics that constitute them, highlighting similarities and different applicabilities. Cryptography is an element that has extreme relevance in data protection and has developed enormously by going on to develop even lightweight and building block encryption techniques. It is important to point out that data that appear secure to us today may not be so tomorrow. This is due to the increase in techniques and tools for collecting data from open sources, as seen in the study presented, and to the advent of quantum computers. In the latter case, classical encryption techniques would fail by exposing our data (Jalali et al., 2019).

A test, in the form of an OSINT survey of the presented tools, was then performed to make a structured comparison between the proposed alternatives.

It emerged from the study how there can be a variety of possible tools that help in research and information gathering, however, the presence and analysis of the human analyst remain essential. The work done by the human operator remains fundamental in their ability to evaluate, judge responses, and link different pieces of information.

## Author contributions

The author confirms being the sole contributor of this work and has approved it for publication.
